# Association of intraoperative hypotension and postoperative acute kidney injury after adrenalectomy for pheochromocytoma: a retrospective cohort analysis

**DOI:** 10.1186/s13741-023-00306-2

**Published:** 2023-05-16

**Authors:** Xia Ruan, Mohan Li, Lijian Pei, Ling Lan, Weiyun Chen, Yuelun Zhang, Xuerong Yu, Chunhua Yu, Jie Yi, Xiuhua Zhang, Yuguang Huang

**Affiliations:** 1 Department of Anesthesiology, Peking Union Medical College Hospital, Chinese Academy of Medical Sciences and Peking Union Medical College, Beijing, China; 2 State Key Laboratory of Complex Severe and Rare Disease, Peking Union Medical College Hospital, Chinese Academy of Medical Sciences and Peking Union Medical College, Beijing, China; 3 Outcomes Research Consortium, Cleveland, OH USA; 4 Central Research Laboratory, Peking Union Medical College Hospital, Chinese Academy of Medical Sciences and Peking Union Medical College, Beijing, China

**Keywords:** Acute kidney injury, Blood pressure, Pheochromocytoma, Adrenalectomy

## Abstract

**Background:**

Perioperative acute kidney injury (AKI) has been one of the leading causes of morbidity and mortality for surgical patients. Pheochromocytoma is a rare, catecholamine-secreting neuroendocrine neoplasm characterized by typical long-term hypertension that needs surgical resection. Our objective was to determine whether intraoperative mean arterial pressures (MAPs) less than 65 mmHg are associated with postoperative AKI after elective adrenalectomy in patients with pheochromocytoma.

**Methods:**

We performed a retrospective review of patients undergoing adrenalectomy for pheochromocytoma between 1991 and 2019 at Peking Union Medical College Hospital, Beijing, China. Two intraoperative phases, before and after tumor resection, were recognized based on distinctly different hemodynamic characteristics. The authors evaluated the association between AKI and each blood pressure exposure in these two phases. The association between the time spent under different absolute and relative MAP thresholds and AKI was then evaluated adjusting for potential confounding variables.

**Results:**

We enrolled 560 cases with 48 patients who developed AKI postoperatively. The baseline and intraoperative characteristics were similar in both groups. Though time-weighted average MAP was not associated with postoperative AKI during the whole operation (OR 1.38; 95% CI, 0.95–2.00;* P* = 0.087) and before tumor resection phase (OR 0.83; 95% CI, 0.65–1.05;* P* = 0.12), both time-weighted MAP and time-weighted percentage changes from baseline were strongly associated with postoperative AKI after tumor resection, with OR 3.50, 95% CI (2.25, 5.46) and 2.03, 95% CI (1.56, 2.66) in the univariable logistic analysis respectively, and with OR 2.36, 95% CI (1.46, 3.80) and 1.63, 95% CI (1.23, 2.17) after adjusting sex, surgical type (open vs. laparoscopic) and estimated blood loss in the multiple logistic analysis. At any thresholds of MAP less than 85, 80, 75, 70, and 65 mmHg, prolonged exposure was associated with increased odds of AKI.

**Conclusions:**

We found a significant association between hypotension and postoperative AKI in patients with pheochromocytoma undergoing adrenalectomy in the period after tumor resection. Optimizing hemodynamics, especially blood pressure after the adrenal vessel ligation and tumor is resected, is crucial for the prevention of postoperative AKI in patient with pheochromocytoma, which could be different from general populations.

## Background

Perioperative morbidity and mortality continue to be a major health burden in current medical practice (Bartels, et al. 2013; Goren and Matot 2015; Wijnberge, et al. 2021). Among different types of postoperative organ dysfunction, acute kidney injury (AKI) is especially noticeable. The incidence of AKI could be as high as 20 to 40% in high-risk patients and may further affect other organ systems, increasing the risk of multiorgan failure and death (Bauerle, et al. 2011). Ongoing efforts are needed in perioperative medicine to find early detection strategies for AKI and better approaches to prevent kidney injury. Large cohort studies have reported that intraoperative mean arterial pressures (MAPs) less than 65 mmHg are associated with postoperative AKI in patients recovering from noncardiac surgery (Ahuja, et al. 2020; Loffel, et al. 2020; Mathis, et al. 2020; Walsh, et al. 2013). However, whether this specific MAP threshold of 65 mmHg is still applicable for patients with long-term hypertension whose autoregulatory pressure-organ perfusion curve moves towards the right-hand side remains uncertain (Asfar, et al. 2014; Hill, et al. 2010; Iversen, et al. 1987; Moman, et al. 2018).

Pheochromocytoma is a rare, catecholamine-secreting neuroendocrine neoplasm arising from chromaffin cells in the adrenal medulla (Farrugia and Charalampopoulos 2019). Surgical resection is often the first-line treatment (Toniato, et al. 2007). Patients with pheochromocytoma often have severe long-term secondary hypertension (Pappachan, et al. 2018). Even after adequate preoperative drug preparation at least 7 to 10 days before the surgical procedure, substantial intraoperative hemodynamic instability is expected in patients with pheochromocytoma resection, regardless of preoperative hormonal activity level (Weingarten, et al. 2010), while precipitous hypotension is common after adrenal vessels ligation and tumor removal (Weingarten, et al. 2017). Approximately 30% of the patients suffered from hypotension intraoperatively, 50% need vasopressors and 10% developed postoperative AKI eventually (Kinney, et al. 2000). However, there is little evidence elucidating the association between intraoperative hypotension and AKI in patients diagnosed with pheochromocytoma.

In order to find better perioperative management strategies to prevent postoperative kidney injury in this typical population characterized by chronic hypertension and unstable intraoperative hemodynamic profile, we investigated a cohort of patients with pheochromocytoma that received surgical resection to evaluate the relationship between intraoperative blood pressure and postoperative AKI. Our main hypothesis is that MAP less than 65 mmHg is associated with postoperative AKI in patients with pheochromocytoma. We also aimed to explore an optimized intraoperative target blood pressure to prevent AKI in patients with pheochromocytoma.

## Methods

### Study cohort

**Fig. 1:**
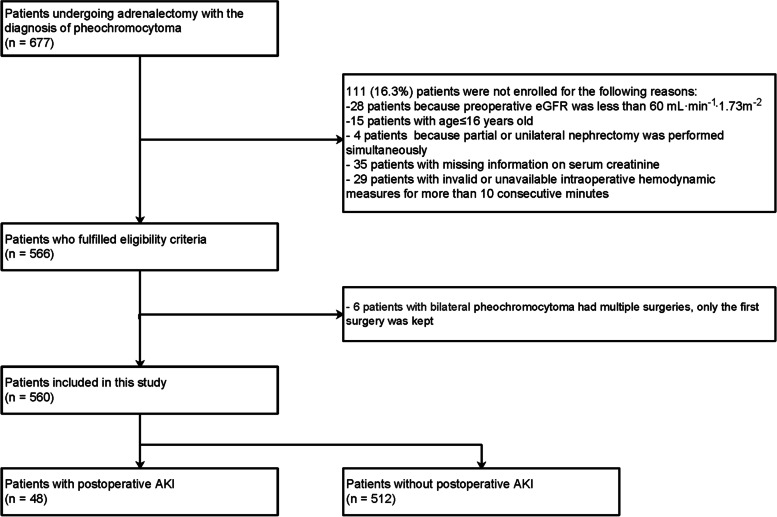
Participant flowchart. AKI, acute kidney injury; eGFR, estimated glomerular filtration rate

The study included patients undergoing adrenalectomy with a diagnosis of pheochromocytoma between 1991 and 2019 at Peking Union Medical College Hospital, Beijing, China. All patients were diagnosed with pheochromocytoma based on postoperative pathological reports. The exclusion criteria include (1) patients with chronic kidney disease defined as a preoperative estimated glomerular filtration rate (eGFR) less than 60 mL·min^−1^·1.73 m^−2^ or patients who were on dialysis, (2) patients aged ≤ 16 years old, (3) patients with partial or unilateral nephrectomy simultaneously, (4) patients with missing information for preoperative or postoperative serum creatinine, and (5) patients with invalid or unavailable intraoperative hemodynamic data for more than 10 consecutive minutes. If patients with bilateral pheochromocytoma had multiple surgeries, only the first surgery was kept. A patient inclusion/exclusion flow diagram is presented in Fig. 1.

### Perioperative management

All patients diagnosed with pheochromocytoma via biochemical tests and imaging examinations were treated with nonselective α-adrenergic blockade (phenoxybenzamine) or selective α-adrenergic blockade (prazosin, doxazosin, or terazosin) at least 2 weeks before the operation. The target blood pressure was lower than 120/80 mmHg when sitting, with systolic blood pressure (SBP) no less than 90 mmHg while standing. Metoprolol was added to control episodes of tachycardia if necessary with a heart rate (HR) goal of 60 to 80 beats/min. When blood pressure control was inadequate or in patients with intolerable side effects, calcium channel blockers or angiotensin-converting enzyme (ACE) inhibitors were added. All patients underwent surgery with general anesthesia and used arterial catheter to monitor blood pressure continuously. During the operation, substantial rises in blood pressure were controlled by bolus or continuous infusion of phentolamine, sodium nitroprusside, or urapidil, and the hypotension after tumor resection was treated by continuous infusion of vasopressors (e.g., norepinephrine and/or epinephrine) and fluid infusion. All treatments were decided at the discretion of the anesthesiologist to keep blood pressure and heart rate stable. Patients were transferred to the intensive care unit after surgery and would be transferred back to floor wards when mechanical ventilation and vasopressors were no longer needed.

### Data source

The study was approved by the Institutional Review Board of Peking Union Medical College Hospital prior to data extraction. Given the retrospective nature of the study, the requirement of written informed consent was waived. Encounter-level patient data (demographics), comprehensive time-stamped medication orders, medication records, laboratory results, admission and discharge diagnoses (International Classification of Diseases Ninth Revision Clinical Modification [ICD-9-CM codes]), and procedures performed were extracted from the hospital information system (DHC Software Co., Ltd, Beijing, China) between 2013 and 2019, or collected by the study team via retrospective chart review between 1991 and 2012.

Preoperative serum creatinine was defined as the serum creatinine obtained in closest proximity to the date of surgery. Peak postoperative serum creatinine was defined as the highest creatinine level obtained during the 7 days after surgery. Internal auditing was performed to ensure data accuracy. Preoperative kidney function was characterized according to the patient’s estimated glomerular filtration rate (eGFR) using the Chronic Kidney Disease Epidemiology Collaboration (CKD-EPI) equation (Levey, et al. 2009). Preoperative hemoglobin was recorded as the hemoglobin concentration taken closest to the time before surgery. Postoperative hemoglobin was measured in the first 24 h after surgery.

Baseline hemodynamic variables, defined as SBP, diastolic blood pressure (DBP), and MAP, which were recorded twice throughout the 24 h before the surgery, were collected by the study team via retrospective chart review using the mean of the two measurements. Intraoperative hemodynamic variables were extracted from anesthesia information management system (Easymonitor Technology Co., Ltd, Beijing, China) between 2013 and 2019, which cannot be modified by clinicians, but can be identified as artifactual. Invasive pressures were recorded at 5-min intervals. We removed artifacts using the following rules, in order: (1) blood pressures documented as artifacts; (2) pressures out-of-range defined by (a) SBP greater than or equal to 300 or SBP less than or equal to 20 mmHg, (b) SBP less than or equal to DBP + 5 mmHg, or (c) DBP less than or equal to 5 mmHg or DBP greater than or equal to 225 mmHg. Between 1991 and 2012, intraoperative hemodynamic variables were collected by the study team via retrospective chart review and were recorded at 5-min intervals. Pressures between measurements were linearly interpolated.

### Statistical analysis

The primary outcome measure of the study was postoperative AKI. We defined AKI according to the KDIGO clinical practice guidelines (Khwaja 2012). In our retrospective study, the hourly urine volume was not recorded in the medical records, and because some studies have disputes about the urine output in KDIGO guidelines (Katabi, et al. 2021), we only used elevated creatinine as the diagnostic basis of AKI. Patients were considered to have AKI if the highest postoperative serum creatinine value was either more than 1.5-fold or more than 0.3 mg/dl greater than the preoperative concentration.

Univariable logistic regression model was used to examine the association between relevant patient demographic data, previous medical history, cardiac medication history, perioperative creatinine, baseline eGFR, intraoperative characteristics, and postoperative AKI. Univariable logistic regression model was also conducted to examine the association between intraoperative hemodynamic variables (including absolute threshold, changes from baseline, and area under the curve (AUC) under different threshold) and postoperative AKI.

Multivariable logistic regression model was constructed to examine the adjusted association between intraoperative hemodynamic variables and postoperative AKI, while accounting for the effects of confounding factors. As adrenalectomy for pheochromocytoma, hypertensive episodes are common before tumor resection because of the excessive catecholamine secretion (i.e., during endotracheal intubation, creation of pneumoretroperitoneum, and manipulation of the tumor), while hypotensive episodes often occur after resection of the pheochromocytoma, intraoperative hemodynamic variables were both calculated during the whole procedure and in 2 separate phases (before tumor resection and after tumor resection). Given the particularity that patients with pheochromocytoma usually suffered from chronic hypertension, the incidence of AKI under different absolute MAP thresholds, including MAP < 65 mmHg and higher MAPs (i.e., MAP < 85, < 80, < 75, < 70 mmHg), as well as different relative decrease from baseline (i.e., 10%, 15%, 20%, 25% and 30%) was then using multivariable logistic regression respectively for further study, aiming to find a more sensitive absolute and relative MAP threshold in patients with pheochromocytoma for postoperative AKI. For each absolute and relative MAP thresholds, we divided the total time duration under this threshold into three values (using 0 min, 1–19 min, and > 20 min considering the sample size in each time group), and constructed an independent multivariable logistic regression model for this specific threshold. Since pheochromocytoma is a rare disease, the sample size of AKI patients included in this study is limited, then the use of Bonferroni correction may lead to a false-negative result. Therefore, we did not apply Bonferroni correction for multiple statistical comparisons, and our result was interpreted in an initial exploratory manner.

All statistical tests were two-tailed at a significance level of 0.05. We used R version 4.0.2 for all statistical analyses.

## Results

### Patient and treatment characteristics

In a total of 677 patients who underwent adrenalectomy with pheochromocytoma between 1991 and 2019, the analysis included 560 patients who met our inclusion and exclusion criteria (Fig. 1). Forty-eight patients were diagnosed as postoperative AKI, including 43 at stage 1, 3 at stage 2, and 2 at stage 3. The overall incidence of postoperative AKI was 8.6%. The perioperative characteristics of patients with and without AKI are summarized in Table 1. The baseline eGFR was well balanced, with 100.6 ± 17.2 mL·min^−1^·1.73 m^−2^ in the AKI group and 100.0 ± 16.2 mL ·min^−1^·1.73 m^−2^ in the non-AKI group. The previous medical history, cardiac medication history, year of surgery, preoperative creatinine, preoperative and postoperative hemoglobin, and baseline hemodynamic variables (SBP, DBP, MAP) were balanced in the postoperative AKI and non-AKI groups.

**Table 1:** Univariable relationship between patient baseline, intraoperative characteristics, and postoperative AKI

Factors	AKI (*n* = 48)	No AKI (*n* = 512)	Unadjusted OR (95% CI)	*P* values*
**Age, yr**	45 ± 13	44 ± 13	1.00 (0.98, 1.02)	0.89
**Male, *n* (%)**	33 (68.8)	242 (47.3)	2.45 (1.30, 4.63)	0.006
**ASA physical status, *n* (%)**			1.13 (0.69, 1.85)	0.62
1	1 (2.1)	45 (8.8)		
2	30 (62.5)	275 (53.7)		
3	17 (35.4)	192 (37.5)		
**Use of arterial catheter, *n* (%)**	48 (100)	512 (100)	NA	NA
**Previous medical history, *n* (%)**				
Coronary artery disease	4 (8.3)	19 (3.7)	2.36 (0.77, 7.24)	0.13
Congestive heart failure	0 (0.0)	14 (2.7)	NA	0.62**
Cerebral vascular disease	4 (8.3)	13 (2.5)	3.49 (1.09, 11.16)	0.035
Diabetes requiring medication	15 (31.2)	116 (22.7)	1.55 (0.81, 2.96)	0.18
**Cardiac medication history, *n* (%)**				
α-blocker	48 (100.0)	503 (98.2)	NA	> 0.99**
ß-blocker	9 (18.8)	108 (21.1)	0.86 (0.41, 1.84)	0.70
CA blocker	11 (22.9)	58 (11.3)	2.33 (1.13, 4.81)	0.023
ACE inhibitor/Angiotensin receptor blocker	4 (8.3)	15 (2.9)	3.01 (0.96, 9.47)	0.059
**Preoperative**				
Preoperative medication time, day	40 [25, 48]	38 [26, 53]	1.00 (0.99, 1.01)	0.41
Preoperative hemoglobin, g/L	139 ± 15	136 ± 16	1.01 (0.99, 1.03)	0.18
Preoperative creatinine, μmol/L	74.4 ± 16.3	71.4 ± 15.4	1.01 (0.99, 1.03)	0.20
**Baseline eGFR, ml·min·1.73 m**^**−2**^	100.6 ± 17.2	100.0 ± 16.2	1.00 (0.98, 1.02)	0.80
Baseline MAP	94 ± 11	90 ± 11	1.03 (0.99, 1.06)	0.035
**Intraoperative**				
Surgical time, min	178 [133, 216]	110 [87, 144]	1.02 (1.01, 1.02)	< 0.001
Open surgery, *n* (%)	24 (50.0)	82 (16.0)	5.24 (2.84, 9.68)	< 0.001
Estimated blood loss, ml^a^	1000 [375, 2500]	100 [50, 400]	2.53 (1.97, 3.25)	< 0.001
Red blood cells transfusion				
Not received, *n* (%)	31 (64.6)	448 (87.5)	Ref = 1	< 0.001
Received, *n* (%)	17 (35.4)	64 (12.5)	3.84 (2.01, 7.33)	
Total red blood cells transfusion, ml	1600 [800, 2750]	800 [400, 1018]		
Total crystalloids, ml^a^	2550 [2000, 3500]	2500 [2000, 3000]	4.67 (2.03, 10.73)	< 0.001
Total colloids, ml^a^	1000 [500, 2125]	1000 [500, 1500]	4.67 (2.03, 10.74)	< 0.001
Vasoactive drugs after tumor resection, *n* (%)				0.15
None	15 (31.2)	199 (38.9)	Ref = 1	
Vasopressors only	25 (52.1)	272 (53.1)	1.22 (0.63, 2.37)	
Other	8 (16.7)	41 (8.0)	2.59 (1.03, 6.51)	
Vasodilators only	7 (14.6)	38 (7.4)		
Vasopressors + vasodilators	1 (2.1)	3 (0.6)		
Postoperative Hemoglobin (g/L)	110 ± 18	114 ± 15	0.98 (0.96, 1.00)	0.060
**Diagnosis, *n* (%)**				0.57
Unilateral pheochromocytoma	43 (89.6)	444 (86.7)	Ref = 1	
Other	5 (10.4)	68 (13.3)	0.76 (0.29, 1.98)	
Bilateral pheochromocytoma	2 (4.2)	28 (5.5)		
MEN syndrome	3 (6.2)	32 (6.2)		
VHL syndrome	0 (0.0)	8 (1.6)		
**Tumor size, cm**	6 ± 3	5 ± 2	1.32 (1.16, 1.49)	< 0.001
**Year of surgery, *n* (%)**				0.063
1991–2000	8 (16.7)	37 (7.2)	Ref = 1	
2001–2010	14 (29.2)	126 (24.6)	0.51 (0.20, 1.32)	
2011–2020	26 (54.2)	349 (68.2)	0.34 (0.15, 0.82)	

Data are presented as mean ± SD, median [25th, 75th percentiles] or *n* (%)

*ACE* Angiotensin-converting enzyme, *AKI* Acute kidney injury, *ASA* American Society of Anesthesiologists, *CA* Calcium channel, *eGFR* Estimated glomerular filtration rate, *MAP* Mean arterial pressure, *MEN* Multiple endocrine neoplasia, *VHL* Von Hippel-Lindau

^*^P values from univariable logistic regression; ** from Fisher’s exact test

^a^Log-transformed estimated blood loss, total crystalloids, or total colloid

### Primary adjusted outcomes

Univariable analyses showed that patients who developed postoperative AKI had larger tumor size, more proportion of male, received open surgery, longer operation time, more estimated blood loss, more red blood cells transfusion, and more intraoperative crystalloids and colloids infusion (all* P* < 0.001; Table 1). Time-weighted average MAP was not associated with postoperative AKI during the whole operation (OR 1.38; 95% CI, 0.95–2.00;* P* = 0.087) and before tumor resection phase (OR 0.83; 95% CI, 0.65–1.05;* P* = 0.12). However, after tumor resection, patients who developed postoperative AKI had lower MAP, greater % MAP decreases from baseline, and more cumulated minutes under all thresholds (MAP < 85, 80, 75, 70, and 65 mmHg) compared to those with no evidence of AKI (all* P* < 0.001; Table 2). Hypotension was strongly associated with postoperative AKI which could only be observed in the period after tumor resection.

**Table 2:** Univariable relationship between intraoperative hemodynamic variables and postoperative AKI

Exposures	AKI (*n* = 48)	No AKI (*n* = 512)	Unadjusted OR (95% CI)	*P* values*
**Baseline**				
Baseline SBP, mmHg	125 ± 15	122 ± 14	1.02 (1.00, 1.04)	0.11
Baseline DBP, mmHg	78 ± 11	75 ± 11	1.03 (0.99, 1.06)	0.024
Baseline MAP, mmHg	94 ± 11	90 ± 11	1.03 (0.99, 1.06)	0.035
**Intraoperative hemodynamic variables**				
Time-weighted average SBP				
SBP^a^, mmHg	121 ± 14	125 ± 12	1.38 (1.06, 1.81)	0.017
% SBP change from baseline^b^, %	− 3 ± 16	4 ± 15	1.36 (1.09, 1.68)	0.005
Time-weighted average DBP				
DBP^a^, mmHg	69 ± 9	71 ± 8	1.21 (0.83, 1.76)	0.327
% DBP change from baseline^b^, %	− 10 ± 16	− 4 ± 17	1.27 (1.04, 1.56)	0.019
Time-weighted average MAP				
MAP^a^, mmHg	87 ± 10	89 ± 8	1.38 (0.95, 2.00)	0.087
% MAP change from baseline^b^, %	− 7 ± 15	− 1 ± 15	1.37 (1.10, 1.72)	0.006
AUC under MAP^c^, mmHg·min				
MAP < 85	584 [325, 1046]	291 [121, 497]	1.20 (1.13, 1.28)	< 0.001
MAP < 80	350 [177, 751]	115 [31, 267]	1.34 (1.22, 1.47)	< 0.001
MAP < 75	173 [91, 491]	29 [0, 115]	1.62 (1.40, 1.87)	< 0.001
MAP < 70	67 [17, 327]	0 [0, 32]	2.21 (1.74, 2.82)	< 0.001
MAP < 65	18 [0, 166]	0 [0, 0]	4.25 (2.68, 6.75)	< 0.001
**Before tumor resection**				
Time-weighted average SBP				
SBP^a^, mmHg	138 ± 18	135 ± 17	0.89 (0.75, 1.05)	0.16
% SBP change from baseline^b^, %	12 ± 19	12 ± 19	1.01 (0.86, 1.18)	0.95
Time-weighted average DBP				
DBP^a^, mmHg	78 ± 11	76 ± 10	0.83 (0.63, 1.09)	0.17
% DBP change from baseline^b^, %	1 ± 17	4 ± 19	1.07 (0.91, 1.26)	0.42
Time-weighted average MAP				
MAP^a^, mmHg	98 ± 12	96 ± 12	0.83 (0.65, 1.05)	0.12
% MAP change from baseline^b^, %	6 ± 16	7 ± 17	1.05 (0.88, 1.25)	0.59
AUC under MAP^c^, mmHg·min				
MAP < 85	60 [4, 150]	42 [0, 156]	1.08 (0.97, 1.20)	0.17
MAP < 80	13 [0, 94]	8 [0, 69]	1.16 (0.99, 1.34)	0.061
MAP < 75	0 [0, 36]	0 [0, 19]	1.33 (1.05, 1.69)	0.017
MAP < 70	0 [0, 9]	0 [0, 0]	1.62 (1.11, 2.37)	0.012
MAP < 65	0 [0, 0]	0 [0, 0]	2.19 (1.17, 4.11)	0.015
**After tumor resection**				
Time-weighted average SBP				
SBP^a^, mmHg	103 ± 15	116 ± 11	3.30 (2.29, 4.77)	< 0.001
% SBP change from baseline^b^, %	− 17 ± 15	− 4 ± 15	2.22 (1.69, 2.92)	< 0.001
Time-weighted average DBP				
DBP^a^, mmHg	61 ± 9	66 ± 8	2.24 (1.49, 3.37)	< 0.001
% DBP change from baseline^b^, %	− 21 ± 15	− 11 ± 16	1.63 (1.29, 2.06)	< 0.001
Time-weighted average MAP				
MAP^a^, mmHg	75 ± 10	82 ± 8	3.50 (2.25, 5.46)	< 0.001
% MAP change from baseline MAP^b^, %	− 20 ± 14	− 8 ± 14	2.03 (1.56, 2.66)	< 0.001
AUC under MAP^c^, mmHg·min				
MAP < 85	512 [271, 746]	206 [64, 352]	1.45 (1.31, 1.62)	< 0.001
MAP < 80	304 [144, 519]	81 [10, 189]	1.65 (1.44, 1.90)	< 0.001
MAP < 75	154 [69, 358]	15 [0, 79]	2.09 (1.70, 2.57)	< 0.001
MAP < 70	51 [6, 235]	0 [0, 17]	3.10 (2.20, 4.38)	< 0.001
MAP < 65	9 [0, 117]	0 [0, 0]	8.20 (4.05, 16.59)	< 0.001

Data are presented as mean ± SD, median [25th, 75th percentiles]

*AKI*, Acute kidney injury, *SBP*, Systolic arterial pressure, *DBP*, Diastolic arterial pressure, *MAP*, Mean arterial pressure, *HR*, Heart rate, *AUC*, Area under the curve

^*^P values from univariable logistic regression

^a^Odds ratio was estimated per 10 units decrease in SBP, DBP, MAP, and HR

^b^Odds ratio was estimated per 10% decrease from baseline in SBP, DBP, MAP, and HR

^c^Odds ratio was estimated per 10 unit decrease of MAP every 10 min. For example, OR = 1.45 for AUC under MAP < 85 mmHg after tumor resection, that is, the odds for developing postoperative AKI would increase by 45% for each 10 mmHg decrease in MAP every 10 min

**Fig. 2:**
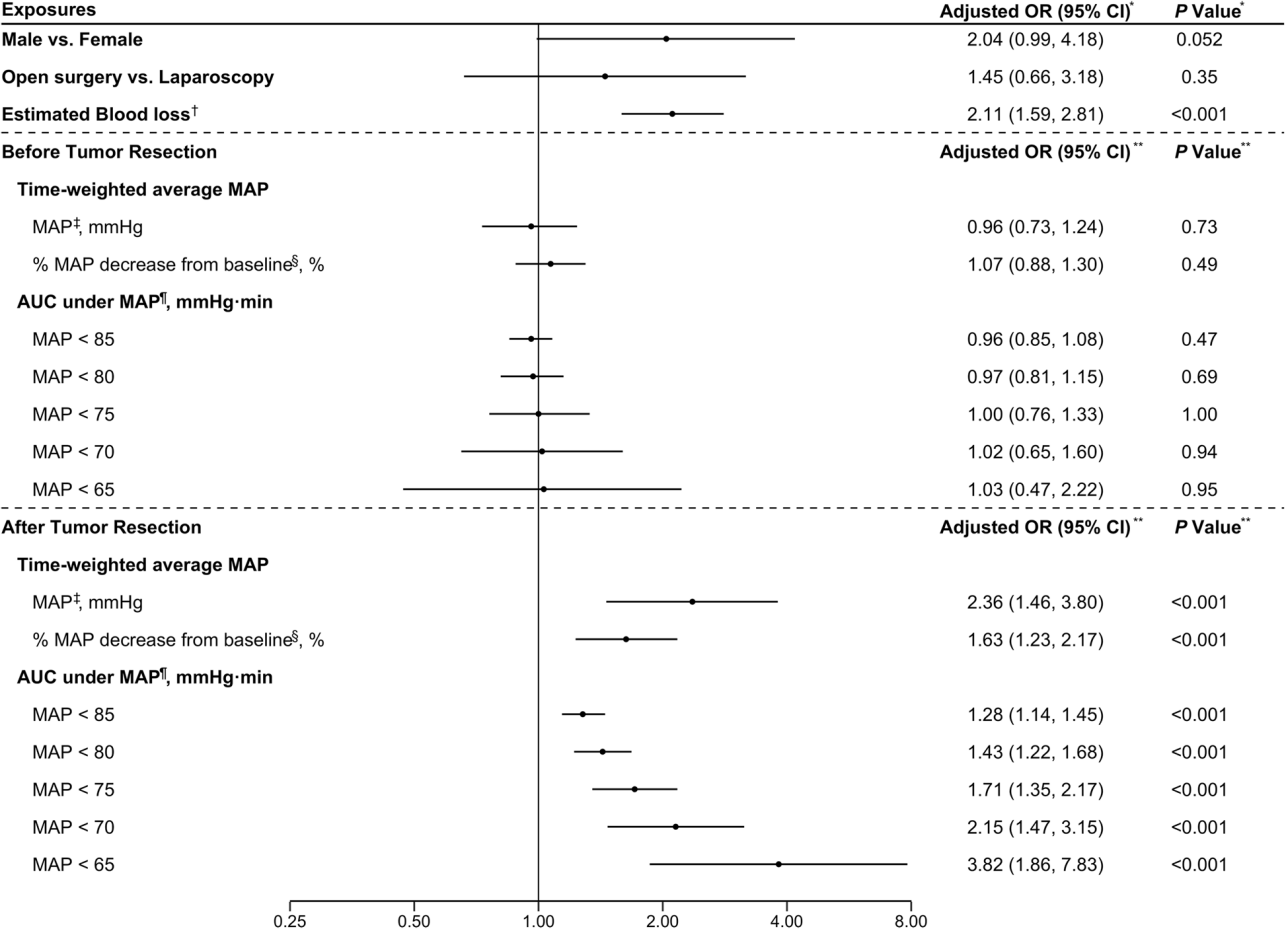
Multivariable relationship between intraoperative MAP exposures and postoperative AKI. AKI, acute kidney injury; MAP, mean arterial pressure; HR, heart rate; AUC, area under the curve. *OR and *P* values from multivariable logistic regression using sex, surgical procedure type, log-transformed estimated blood loss, and time-weight MAP percent change after tumor resection as variables. **Multivariable logistic regression models were adjusted for sex, surgical procedure type, and log-transformed estimated blood loss. ^†^Log transformed estimated blood loss. ^‡^Odds ratio was estimated per 10 units decrease in MAP. ^§^Odds ratio was estimated per 10% decrease from baseline in MAP. ^¶^Odds ratio was estimated per 10 unit decrease of MAP every 10 min. For example, OR = 1.28 for AUC under MAP < 85 mmHg after tumor resection, that is, the odds for developing postoperative AKI would increase by 28% for each 10 mmHg decrease in MAP every 10 min

The correlation coefficients were obtained using the Spearman correlation method, showing strong collinearity between operation time, blood transfusion, fluid infusion, and estimated blood loss. Considering blood loss is the reason for others, we used estimated blood loss in the final model. Multivariable analyses showed that in the period after tumor resection, both intraoperative time-weighted average MAP (OR 2.36; 95% CI, 1.46–3.80; *P* < 0.001) and % MAP decreases from baseline (OR 1.63; 95% CI, 1.23–2.17; *P* = 0.001) were associated with a statistically significant increase in postoperative AKI. The AUC for MAP < 85, 80, 75, 70, and 65 mmHg were also associated with statistically significant increase in postoperative AKI (OR 1.28, 1.43, 1.71, 2.15, 3.82 respectively, *P* < 0.001; Fig. 2), after adjusting for sex, surgical type (open vs. laparoscopy), and estimated blood loss. We have also tested fluid transfusion and operation time as confounding factors via sensitivity analyses, and the primary outcome remained similar.

### Relationship between exposure categories and outcome

Multivariable analyses showed that in the period after tumor resection, time spent under the absolute threshold of MAP less than < 65 mmHg had increased odds of AKI. Compared with patients that had never experienced a MAP less than 65 mmHg, those with longer periods of a MAP less than 65 mmHg had significantly increased odds of AKI, (OR 4.43; 95% CI, 2.09–9.35; *P* < 0.001 for less than 20 min vs OR 6.04; 95% CI, 1.75 to 20.89; *P* < 0.001 for more than 20 min; Table 3). In addition, when higher absolute MAP thresholds or relative decrease from baseline were evaluated by multivariable analyses, similar results were obtained that absolute MAP less than 80, 75, and 70 mmHg and relative MAP threshold decrease from baseline more than 30%, 25%, 20%, 15%, and even 10% (all *P* < 0.05; Table 3) had increased odds of AKI, after adjusting for sex, surgical type (open vs. laparoscopy), and estimated blood loss.

**Table 3:** Multivariable association with absolute and relative MAP thresholds after tumor resection and postoperative AKI

Threshold	AKI (*n* = 48)	No AKI (*n* = 512)	Adjusted OR (95% CI)*	*P* values*
Baseline MAP, mmHg	94 ± 11	90 ± 11	1.02 (0.99, 1.05)	0.19
**Time under absolute MAP**				
MAP < 85 mmHg				
0 min, *n* (%)	1 (2.1)	53 (10.4)	Ref	0.050
1–19 min, n (%)	3 (6.3)	95 (18.6)	2.07 (0.20, 21.51)	
≥ 20 min*, n* (%)	44 (91.7)	364 (71.1)	5.21 (0.68, 40.05)	
MAP < 80 mmHg				
0 min, *n* (%)	1 (2.1)	107 (20.9)	Ref	0.004
1–19 min, *n* (%)	11 (22.9)	157 (30.7)	8.88 (1.09, 72.42)	
≥ 20 min, *n* (%)	36 (75.0)	248 (48.4)	11.16 (1.46, 85.10)	
MAP < 75 mmHg				
0 min, *n* (%)	4 (8.3)	201 (39.3)	Ref	< 0.001
1–19 min, *n* (%)	15 (31.3)	192 (37.5)	3.24 (1.02, 10.29)	
≥ 20 min,* n* (%)	29 (60.4)	119 (23.2)	7.94 (2.61, 24.18)	
MAP < 70 mmHg				
0 min, *n* (%)	11 (22.9)	340 (66.4)	Ref	< 0.001
1–19 min, *n* (%)	22 (45.8)	140 (27.3)	4.87 (2.20, 10.80)	
≥ 20 min, *n* (%)	15 (31.3)	32 (6.3)	9.21 (3.37, 25.14)	
MAP < 65 mmHg				
0 min, *n* (%)	20 (41.7)	432 (84.4)	Ref	< 0.001
1–19 min, *n* (%)	19 (39.6)	73 (14.3)	4.43 (2.09, 9.35)	
≥ 20 min, *n* (%)	9 (18.8)	7 (1.4)	6.04 (1.75, 20.89)	
**Time under relative MAP Thresholds**				
MAP decrease from baseline > 10%				
0 min,* n* (%)	3 (6.3)	140 (27.3)	Ref	0.005
1–19 min, *n* (%)	4 (8.3)	106 (20.7)	2.09 (0.44, 9.92)	
≥ 20 min,* n* (%)	41 (85.4)	266 (52.0)	5.01 (1.47, 17.08)	
MAP decrease from baseline > 15%				
0 min, *n* (%)	3 (6.3)	201 (39.3)	Ref	< 0.001
1–19 min, *n* (%)	11 (22.9)	125 (24.4)	5.97 (1.57, 22.71)	
≥ 20 min, *n* (%)	34 (70.8)	186 (36.3)	7.86 (2.29, 26.94)	
MAP decrease from baseline > 20%				
0 min, *n* (%)	6 (12.5)	262 (51.2)	Ref	< 0.001
1–19 min, *n* (%)	12 (25.0)	120 (23.4)	3.22 (1.12, 9.25)	
≥ 20 min, *n* (%)	30 (62.5)	130 (25.4)	6.63 (2.56, 17.14)	
MAP decrease from baseline > 25%				
0 min, *n* (%)	7 (14.6)	339 (66.2)	Ref	< 0.001
1–19 min, *n* (%)	23 (47.9)	106 (20.7)	8.77 (3.50, 21.98)	
≥ 20 min, *n* (%)	18 (37.5)	67 (13.1)	6.53 (2.42, 17.66)	
MAP decrease from baseline > 30%				< 0.001
0 min, *n* (%)	18 (37.5)	406 (79.3)	Ref	
1–19 min, *n* (%)	16 (33.3)	80 (15.6)	3.63 (1.67, 7.88)	
≥ 20 min, *n* (%)	14 (29.2)	25 (4.9)	5.36 (2.09, 13.75)	

*AKI*, acute kidney injury, *MAP*, mean arterial pressure

^*^P values from multivariable logistic regression. Multivariable logistic regression models were adjusted for sex, surgical procedure type, and log-transformed estimated blood loss

## Discussion

Targeted blood pressure during the perioperative period of noncardiac surgery to prevent postoperative AKI is becoming a research hotspot. We designed this cohort study to examine the associations between intraoperative blood pressures and postoperative AKI in patients with pheochromocytoma and aimed to find better intraoperative management strategies to prevent kidney injury. Our results revealed that during the period after tumor resection, time spent under the absolute threshold of MAP less than < 65 mmHg increased odds of AKI in this population, which are generally consistent with previous studies reporting that the risk of postoperative AKI increases when MAP is lower than 55–65 mmHg (Ahuja, et al. 2020; Loffel, et al. 2020; Salmasi, et al. 2017; Sun, et al. 2015; Walsh, et al. 2013).

Similar to other studies, we found that the intraoperative hypotension was associated with increased odds of postoperative AKI during the entire operation in patients diagnosed with pheochromocytoma. These patients usually experience hypertension due to excessive catecholamine secretion, while the dramatic reduction of catecholamine after tumor resection may lead to hypotension episodes. As a result, we divided the whole surgery procedure into two phases (before tumor resection and after tumor resection) according to the pathophysiological and intraoperative hemodynamic characteristics of pheochromocytoma. We found that pre-resection hypotension is less relevant with postoperative AKI than post-resection hypotension which could be explained by the pathophysiological mechanism that hypotension is considered a relatively rare situation before tumor resection than after resection. That is, for the prevention of AKI, anesthesiologists should pay more attention to the timely and effective treatment of post-resection hypotension.

In our study, the AKI group had double the proportion of patients receiving vasodilators (14.6% vs 7.4%) after tumor resection; however, most of the patients who received vasodilators maintained an MAP greater than 65 mmHg. As all treatments were decided by the anesthesiologist to keep blood pressure and heart rate stable, this may be due to the target blood pressure varies in different anesthesiologists (Bijker, et al. 2007). Therefore, we tried to explore optimized management of blood pressure for these patients. When analyzing the cumulative effect of lower blood pressure over time, we found that the AUC was significantly associated with AKI not only when MAP < 65 but also when MAP < 70, 75, 80, and 85 mmHg. It was also worth noting that after tumor resection, in the AKI group, only 9 (18.8%) cases suffered MAP < 65 mmHg and 14 (29.2%) cases suffered relative MAP threshold decreased from baseline > 30%; however, nearly 29 (60.4%) cases suffered MAP < 75 mmHg and 30 (62.5%) cases suffered relative MAP threshold decreased from baseline > 20%, which lasted for at least 20 min. Whereas in the non-AKI group, the proportion was 1.4% and 4.9%, 23.2% and 25.4% respectively. Our findings suggested that for patients with pheochromocytoma suffering from long-term hypertension, it may not be enough to maintain MAP above 65 mmHg during surgery. Given that patients’ MAP could be managed during surgery, we recommended 75 mmHg and 20% as absolute and relative thresholds since more patients with hypotension could be identified under these 2 thresholds without overtreatment especially after tumor resection.

Our study also showed larger tumor size, greater proportion of patients undergoing open surgery and longer duration of surgery in the AKI group than in the non-AKI group, and all these factors may lead to an increase in blood loss. In our study, blood loss was significantly associated with AKI, which was similar to the findings of previous studies (Abar, et al. 2018; Ahuja, et al. 2020). In the context of pheochromocytoma, our center adopted an aggressive blood transfusion approach. Therefore, the postoperative hemoglobin level was the same in both groups, although more blood loss was observed in the AKI group. This result reduced the influence of massive blood loss and lower hemoglobin levels on AKI, but it could not exclude the impact of blood transfusion on AKI, as described by Iyigun et al. (Iyigun, et al. 2019), which is a confounding factor in our study.

There are several limitations of our study. Firstly, our study analyzed data from only a single center, with a limited number of cases, which may have reduced the robustness and generalizability of our findings. Besides, our patients were a highly selected group who suffered from secondary hypertension resulting from a rare disease. We need to be cautious about applying our findings to patients suffering from the more prevalent essential hypertension, and further research is needed. Finally, our study is an initial exploratory about optimized hemodynamic management; well-powered and well-designed studies are required in the future to get to a further conclusion.

## Conclusions

We found a significant association between hypotension and postoperative AKI in patients with pheochromocytoma undergoing adrenalectomy in the period after tumor resection. Patients who developed postoperative AKI had lower MAP and more cumulated minutes under all thresholds (MAP < 85, 80, 75, 70, and 65 mmHg) compared to those with no evidence of AKI. Optimizing hemodynamics, especially blood pressure after the adrenal vessel ligation and tumor is resected, is crucial for the prevention of postoperative AKI in patient with pheochromocytoma, which could be different from general populations.

## Data Availability

The dataset used and analyzed during the current study are available from the corresponding author on reasonable request.

## Abbreviations

AKI: Acute kidney injury
MAP: Mean arterial pressure
SBP: Systolic blood pressure
DBP: Diastolic blood pressure
HR: Heart rate
AUC: Area under the curve
ACE: Angiotensin-converting enzyme
ASA: American Society of Anesthesiologists
CA: Calcium channel
eGFR: Estimated glomerular filtration rate
MEN: Multiple endocrine neoplasia
VHL: Von Hippel-Lindau
